# An Integrated Peptidomics and In Silico Approach to Identify Novel Anti-Diabetic Peptides in Parmigiano-Reggiano Cheese

**DOI:** 10.3390/biology10060563

**Published:** 2021-06-21

**Authors:** Serena Martini, Lisa Solieri, Alice Cattivelli, Valentina Pizzamiglio, Davide Tagliazucchi

**Affiliations:** 1 Department of Life Sciences, University of Modena and Reggio Emilia, 42100 Reggio Emilia, Italy; serena.martini@unimore.it (S.M.); lisa.solieri@unimore.it (L.S.); alice.cattivelli@unimore.it (A.C.); 2 Consorzio del Formaggio Parmigiano Reggiano, 42124 Reggio Emilia, Italy; pizzamiglio@parmigianoreggiano.it

**Keywords:** bioactive peptides, type-2-diabetes, enzyme inhibition, mass spectrometry, Parmigiano-Reggiano, peptidomics, integrated screening method

## Abstract

**Simple Summary:**

Dietary habits and sedentary lifestyle are widely established to be the major risk factors for the development of long-term chronic conditions, such as type-2 diabetes (T2D). In this framework, where consumer’s food choices are even more influenced by an ever-growing awareness on nutritional, environmental, and healthy aspects, the presence and identification of natural bioactive compounds are gaining increasing attention in the scientific community. Pharmacological treatment of T2D is based on the administration of molecules able to inhibit some key enzymes involved in the carbohydrate digestion and insulin secretion. The multiple side-effects of synthetic inhibitors led to an increased demand for natural food-derived anti-diabetic agents. The present work offers a new integrated approach for the identification and selection of new bioactive peptides, able to inhibit the key digestive enzymes implicated in the control of blood glucose level. The novelty lies in the development of a new, quick, and cost-effective integrated methodology supported by confirmed in vitro evidence. In fact, the present work successfully accomplished the identification of two selected candidates with a possible application in the diabetes management. Indeed, the functional and healthy role of Parmigiano-Reggiano cheese in human nutrition was assessed, highlighting its potential anti-diabetic properties.

**Abstract:**

Inhibition of key metabolic enzymes linked to type-2-diabetes (T2D) by food-derived compounds is a preventive emerging strategy in the management of T2D. Here, the impact of Parmigiano-Reggiano (PR) cheese peptide fractions, at four different ripening times (12, 18, 24, and 30 months), on the enzymatic activity of α-glucosidase, α-amylase, and dipeptidyl peptidase-IV (DPP-IV) as well as on the formation of fluorescent advanced glycation end-products (fAGEs) was assessed. The PR peptide fractions were able to inhibit the selected enzymes and fAGEs formation. The 12-month-ripening PR sample was the most active against the three enzymes and fAGEs. Mass spectrometry analysis enabled the identification of 415 unique peptides, 54.9% of them common to the four PR samples. Forty-nine previously identified bioactive peptides were found, mostly characterized as angiotensin-converting enzyme-inhibitors. The application of an integrated approach that combined peptidomics, in silico analysis, and a structure–activity relationship led to an efficient selection of 6 peptides with potential DPP-IV and α-glucosidase inhibitory activities. Peptide APFPE was identified as a potent novel DPP-IV inhibitor (IC_50_ = 49.5 ± 0.5 μmol/L). In addition, the well-known anti-hypertensive tripeptide, IPP, was the only one able to inhibit the three digestive enzymes, highlighting its possible new and pivotal role in diabetes management.

## 1. Introduction

Type 2 diabetes (T2D) is a chronic metabolic condition associated with hyperglycemia and insulin resistance, whose incidence is continuously increasing worldwide [1]. Hyperglycemia is deemed the principal pathogenic factor in T2D since high blood glucose levels are implicated in major health complications including cardiovascular diseases, neuropathy, nephropathy, and retinopathy [2,3]. Therefore, management of T2D involves the control of the blood glucose homeostasis through either lifestyle modification (i.e., physical activity and dietary habits) or pharmacological treatments [4,5,6,7]. Typical therapeutic targets to control hyperglycemia in T2D patients are those enzymes involved in the regulation of post-prandial blood glucose levels such as α-amylase, α-glucosidase, and dipeptidyl peptidase-IV (DPP-IV) [6,7].

The enzymes α-amylase and α-glucosidase are both involved in the gastro-intestinal digestion of complex polysaccharides (such as starch and glycogen) or oligo/disaccharides (such as dextrins or maltose). In particular, α-amylase is implicated in the initial breakdown of the α(1→4) glycosidic linkages in starch and glycogen, releasing shorter oligosaccharides such as dextrins, maltotriose and maltose. The complete hydrolysis of oligosaccharides to glucose is carried out by the small intestine brush-border enzyme α-glucosidase, which hydrolyses non-reducing terminal (1→4)-linked alpha-glucose residues. Their activity results in the release of free monosaccharides, which in turn are absorbed by enterocytes and contribute to the post-prandial elevation of blood glucose levels [6]. The inhibition of α-amylase and/or α-glucosidase results in a delay in glucose absorption, diminishing the post-prandial blood glucose concentration [8]. Inhibition of these enzymes is considered a major approach for the management of T2D [9].

The aminopeptidase DPP-IV is an intestinal membrane-bound brush border prolyl-dipeptidase, whose primary action is to hydrolyze and inactivate the incretin hormones known as glucagon-like peptide 1 (GLP-1) and glucose-dependent insulinotropic polypeptide (GIP) [10]. GLP-1 and GIP are able to stimulate insulin secretion and inhibit glucagon release, lowering the post-prandial hyperglycemia [7]. In fact, the inhibition of DPP-IV prolongs the incretins half-life, resulting in enhanced insulin secretion and reduced hyperglycemia [11].

Several pharmacological inhibitors of these enzymes have been developed and used as effective hypoglycemic agents for the control of diabetes; however, these inhibitors have multiple side effects [8,11]. In addition to the obvious side effects related to the gastro-intestinal tract, such as gastrointestinal pain, diarrhea, and flatulence, synthetic DPP-IV inhibitors can also induce flu-like symptoms, skin reaction, and a modest increased risk of acute pancreatitis [8,11]. These side effects limited the use of synthetic inhibitors, leading to an increasing demand for natural food-derived anti-diabetic agents.

Chronic hyperglycemia in T2D patients resulted in the generation and accumulation of the so-called advanced glycation end-products (AGEs). These compounds are formed from the reaction of glucose and the amino groups of proteins (endogenous Maillard reaction), especially long half-life proteins, and are supposed to be the key factors in the progression of micro- and macro-vascular as well as oxidative stress complications in T2D patients [12,13]. Due to the significant role of AGEs in diabetic complications, several therapeutic approaches to reduce them, including the use of AGEs inhibitors, are currently under clinical evaluation [14].

Several recent meta-analysis and review papers evidenced an inverse association between dairy foods intake and the incidence of T2D [15,16]. Moreover, dairy products intake was inversely related with fasting and post-loaded glucose level and insulinemia [17]. The strongest inverse association between dairy products and the incidence of T2D was found for fermented dairy foods such as yogurt and cheese [16,17]. Fermented dairy product consumption is continuously increasing worldwide as they are considered healthy foods with functional properties [18,19]. Among fermented dairy foods, cheese is considered an important reservoir not only of essential nutrients such as fats, proteins, and minerals, but also of compounds with biological activities including bioactive peptides [20,21].

Generally, bioactive peptides have been described as medium or short amino acids sequences already present in foods or released after protein hydrolysis, as for example in the gastro-intestinal tract during food digestion [22]. Several biologically active peptides have been characterized in cheeses exhibiting various activities including angiotensin-converting enzyme (ACE) inhibition, dipeptidyl peptidase-IV (DPP-IV) inhibition, antioxidant activity, mineral-binding activity, and immunomodulatory activity [10,20].

Parmigiano-Reggiano (PR) is a hard cooked cheese made from a mixture of whole and partially skimmed cow milk in combination with natural whey starter [23]. Before marketing, PR undergoes at least 12 months of ripening, during which lactic acid bacteria hydrolyzed caseins release a plethora of different oligopeptides [24]. Very few papers have evidenced the presence of bioactive peptides in PR. Basiricò et al. [25] found 4 ACE-inhibitory peptides in 12-month-ripened PR, whereas, more recently, Solieri et al. [21] identified 40 bioactive peptides (mainly anti-microbial and ACE-inhibitory peptides) in 12-month-ripened PR by high-resolution mass spectrometry. In other study, Martini et al. [26] characterized 26 bioactive peptides in PR samples collected after 12, 18, and 24 months of ripening whose presence and amount was strongly related to the ripening time. However, to the best of our knowledge, no studies have been published about the identification of α-glucosidase or α-amylase inhibitory peptides in cheeses.

Therefore, this study was designed to explore the potential anti-diabetic properties of water-soluble peptides from PR samples at different ripening times by the analysis of the inhibitory activity towards three key metabolic enzymes, i.e., DPP-IV, α-glucosidase and α-amylase, and towards AGE formation. Moreover, a new proposed combined peptidomics and in silico approach was applied to identify new potential inhibitory peptides.

## 2. Materials and Methods

### 2.1. Materials

Enzymes, substrates, and chemicals used for the enzymatic assay and for peptides quantification were from Sigma–Aldrich (Milan, Italy). All MS/MS solvents were supplied by Bio-Rad (Hercules, CA, USA). Ultrafiltration units (Amicon Ultra-4 regenerated cellulose filters) with a molecular weight cut-off of 3 kDa were purchased from Millipore (Milan, Italy). Parmigiano-Reggiano cheese samples at 12 (PR12), 18 (PR18), 24 (PR24), and 30 (PR30) months of ripening were withdrawn from the same cheese factory in the province of Reggio Emilia (Reggio Emilia, Italy). Three slices of about 500 g (height 11 ± 1 cm, radius 22 ± 2 cm) were obtained from the cheese wheels at the different ripening times. Cheese slices were finely ground and immediately stored at -80 °C for subsequent analysis. The production and the ripening processes of Parmigiano-Reggiano cheese, according to the Protected Designation of Origin specifications, are fully described in Tagliazucchi et al. [23]. All the other reagents were from Carlo Erba (Milan, Italy).

### 2.2. Extraction of Water-Soluble Low-Molecular Weight Peptides from Parmigiano-Reggiano Samples

Peptides were extracted according to Sforza et al. [24] with slight modifications. Five grams of grounded PR samples and 45 mL of HCl (0.1 mmol/L) were mixed and homogenized with an Ultra-Turrax homogenizer for 1 min followed by ice-bath intervals of 1 min (3 cycles). After centrifugation (4000 g; 40 min; 4 °C), the supernatants were withdrawn and filtered through paper filters. Low-molecular weight peptides were then extracted by ultra-filtration as detailed in Tagliazucchi et al. [27]. The extractions were carried out in triplicate and the peptide fractions pooled together before the analysis. Water-soluble low-molecular weight peptides were then quantified through the TNBS (2,4,6-trinitrobenzenesulfonic acid) assay according to Adler–Nissen [28]. L-leucine was used as standard reference.

### 2.3. Analysis of the Enzymatic Inhibitory Activity of Water-Soluble Low-Molecular Weight Peptides Extracted form PR Samples

#### 2.3.1. α-Amylase Assay

α-Amylase assay was performed in accordance with McDougall et al. [29] adapted to a microplate reader. For the reaction, 5 μL of 2 U/mL of porcine pancreatic α-amylase (dissolved in sodium phosphate buffer 20 mmol/L pH 6.9 containing 6.7 mmol/L NaCl) were mixed with 45 μL of sodium phosphate buffer or different concentrations of water-soluble low-molecular weight peptide extracts. The mixture was incubated at 37 °C for 20 min before adding 50 μL of 1% starch solution. The reaction was carried out at 37 °C for 10 min and stopped by the addition of dinitrosalicylic acid solution, followed by 15 min boiling in a water bath. After the addition of 450 μL of water, 200 μL of solution was transferred in a 96-well plate and the absorbance was read at 540 nm. Results were expressed as IC_50_, namely the peptides concentration (expressed in mg/mL) able to inhibit the enzymatic activity by 50%. The IC_50_ values were calculated by plotting the peptide concentration (base-10 logarithm) as a function of the percentage of enzyme inhibition.

#### 2.3.2. α-Glucosidase Assay

α-Glucosidase assay was performed as reported in Bellesia et al. [30], adapted to a microplate reader. The reaction was performed by mixing 66.7 μL of potassium phosphate buffer (67 mmol/L, pH 6.8), 3.3 μL of glutathione 3 mmol/L, 20 μL of different concentrations of water-soluble low-molecular weight peptide extracts, and 5 μL of yeast α-glucosidase 0.2 U/mL. After 20 min of incubation at 37 °C, the reaction was started by the addition of 5 μL of *p*-nitrophenyl-glucose 5 mmol/L. After a further 20 min of incubation at 37 °C, the reaction was stopped by the addition of 150 μL of Na_2_CO_3_ 100 mmol/L. The amount of released *p*-nitrophenol was determined by reading at 405 nm with a microplate reader. Results were expressed as IC_50_ as reported above.

#### 2.3.3. Dipeptidyl Peptidase IV (DPP-IV) Assay

DPP-IV assay was performed as described in Tagliazucchi et al. [31]. Briefly, in a 96-well plate, 10 μL of rat intestinal DPP-IV (0.1 U/mL in the assay) were mixed with 145 μL of Tris-HCl buffer (pH 7.0; 0.1 mol/L) and 40 μL of different concentrations of water-soluble low-molecular weight peptide extracts. After 20 min of incubation at 37 °C, the reaction was started by the addition of 5 μL of glycine-proline-*p*-nitroanilide (6.4 mmol/L). The amount of released *p*-nitroanilide was determined after 20 min of incubation at 37 °C by reading at 405 nm in a microplate reader. Results were expressed as IC_50_ as reported above.

### 2.4. Advanced Glycation End-Products (AGEs) Inhibitory Assay

For the glycation experiments, bovine serum albumin (BSA; 50 mg/mL) was incubated with 0.8 mol/L of glucose and variable amounts of water-soluble low-molecular weight peptide extracts for 7 days at 37 °C. The reaction was carried out in 0.1 mol/L of phosphate buffer (pH 7.4; sodium azide 0.012%). After incubation, the amount of fluorescent AGEs was determined with fluorescent at the excitation and emission maxima of 355 and 405 nm, respectively, versus incubated blanks containing BSA alone or BSA and inhibitors [32]. The data were expressed as IC_50_ as reported above.

### 2.5. Identification of Low Molecular Weight Peptides by Ultra-High-Performance Liquid Chromatography/High-Resolution Mass Spectrometry (UHPLC/HR-MS)

Water-soluble low-molecular weight peptide extracts were submitted to UHPLC/HR-MS analysis for peptide identification. Chromatographic separation was carried out with UHPLC (UHPLC Ultimate 3000 separation module, Thermo Scientific, San Jose, CA, USA) equipped with a C18 column (Acquity UPLC HSS C18 reversed phase, 2.1 × 100 mm, 1.8 μm particle size, Waters, Milan, Italy). Mass spectrometry (MS) and tandem MS experiments were performed on a Q Exactive Hybrid Quadrupole-Orbitrap Mass Spectrometer (Thermo Scientific, San Jose, CA, USA). The binary gradient consisted of two mobile phases: (A) H2O/formic acid (99.9:0.1, *v/v*) and (B) acetonitrile. The gradient began at 2% B, and grew to 27% B in 20 min. The mobile phase composition was raised to 90% in 5 min and maintained for an additional 3 min before coming back to the initial conditions. The flow rate was set at 0.5 mL/min. The mass spectrometer parameters were as follows: spray voltage 4.0 kV, capillary temperature 320 °C, sheath gas 55, and auxiliary gas 30. Full MS parameters were: resolution 70000, AGC target 3e6, maximum IT 243 ms, and scan range 300 to 3000 *m/z*. MS/MS parameters were: resolution 17500, AGC target 5e5, maximum IT 80 ms, and isolation window 2.3 *m/z*.

Peptide sequencing was carried out by using MASCOT (Matrix Science, Boston, MA, USA) protein identification software with the following search parameters: enzyme, none; peptide mass tolerance, ± 5 ppm; fragment mass tolerance, ± 0.12 Da; variable modification, oxidation (M), and phosphorylation (ST); maximal number of post-translational modifications permitted in a single peptide, 4. The assignment procedure was confirmed by the manual verification of MS/MS spectra.

### 2.6. In Silico Analysis

#### 2.6.1. Identification of Previously Reported Bioactive Peptides

Peptides in water-soluble low-molecular weight extracts with previously reported biological activities were searched by using the BIOPEP database and the Milk Bioactive Peptides Database (MBPDB) [33,34]. Only peptides with 100% homology to previously demonstrated bioactive peptides were considered.

#### 2.6.2. Identification of New Potential Bioactive Peptides

Peptides identified in the water-soluble low-molecular weight extracts were screened and selected by using the software Peptide Ranker [35]. Only peptides with a score threshold ≥ 0.5 were considered as potential new bioactive peptides. Selected peptides were then subjected to docking analysis by using Pepsite2 for binding site prediction [36]. The following PDB codes were used: 1PIG, for α-amylase, 5NN3, for α-glucosidase, and 1NU6, for DPP-IV. The selection of bioactive peptide was carried out according to the binding significance (*p* < 0.05) and the structural features of peptides. The stability under gastro-intestinal conditions was carried out in silico by using PeptideCutter software.

Selected peptides were then synthesized (Bio-Fab Research, Rome, Italy) and tested for their inhibitory activity against DPP-IV, α-glucosidase, and α-amylase. The assays were carried out as reported in Section 2.3. The results were expressed as IC_50_ values.

**Figure 1:**
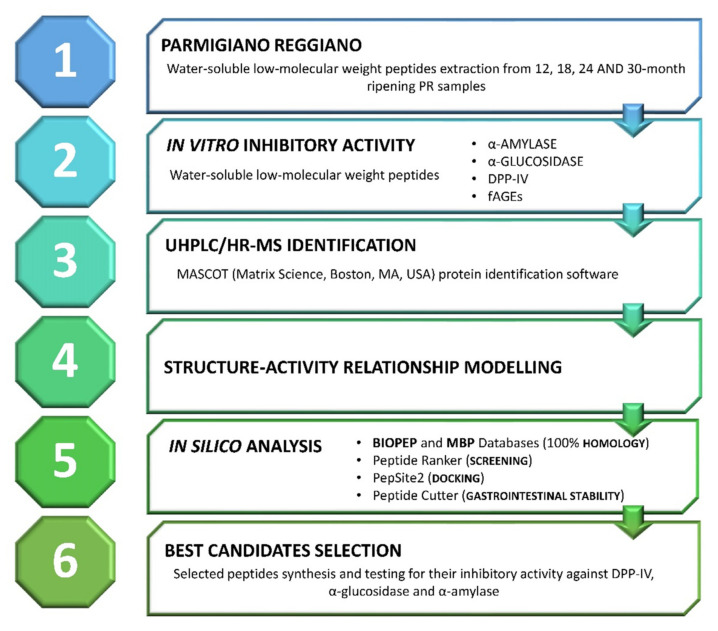
Graphical summary of the proposed study design. The different steps of the present study are summed up to provide an easy and quick overall view of the methodological approach. PR: Parmigiano-Reggiano; DPP-IV: dipeptidyl peptidase IV; fAGEs: fluorescent advanced glycation end-products.

A graphical summary of the described workflow is reported in Figure 1.

### 2.7. Statistical Analysis

All data are showed as mean ± standard deviation (SD) for three replicates for each prepared sample. Univariate analysis of variance (ANOVA) with Tukey post hoc test was applied using GraphPad Prism 6.0 (GraphPad Software, San Diego, CA, USA). The differences were considered significant with *p* < 0.05.

## 3. Results and Discussion

### 3.1. Inhibitory Effect of Water-Soluble Low-Molecular Weight Peptide Extracts of Parmigiano-Reggiano (PR) Samples on α-Amylase, α-Glucosidase, and Dipeptidyl Peptidase-IV (DPP-IV) Activities and Fluorescent Advanced Glycation End-Product (AGEs) Formation

Water-soluble low-molecular weight peptides extracted from PR samples at different ripening stages were tested for their ability to inhibit the activity of three key enzymes (α-amylase, α-glucosidase, and DPP-IV) considered the biological targets for anti-diabetic drugs.

The concentration of peptides in the water-soluble low-molecular weight extracts slightly increased during the first 24 months of ripening passing from 63.54 ± 1.10 mg/g of cheese in PR12 sample to 64.93 ± 1.35 mg/g of cheese in PR18 sample and 81.72 ± 1.10 mg/g of cheese in PR24 sample. After that, the amount of peptides decreased to 76.70 ± 1.35 mg/g of cheese in PR30 sample.

**Figure 2:**
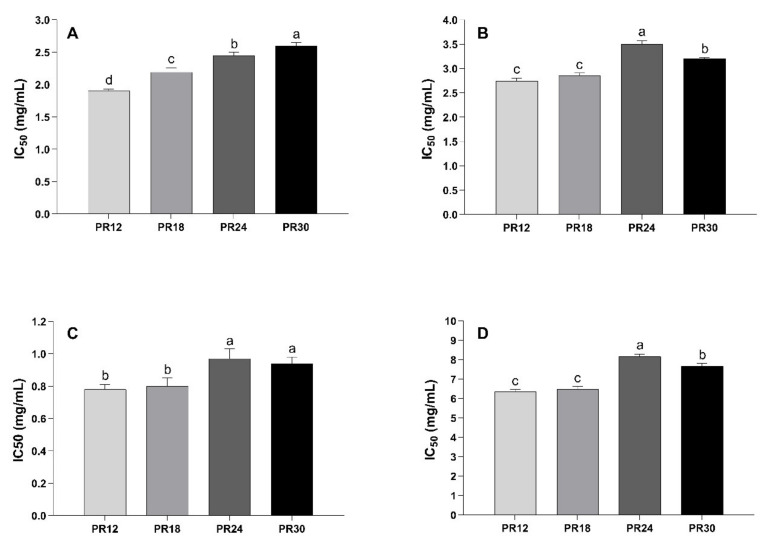
Effect of Parmigiano-Reggiano (PR) water-soluble peptide extracts on the activity of key metabolic enzymes and on advanced glycation end-products formation. (**A**) Inhibition of a-amylase activity. (**B**) Inhibition of a-glucosidase activity. (**C**) Inhibition of dipeptidyl peptidase IV activity. (**D**) Inhibition of fluorescent advanced glycation end-products formation. Water-soluble peptide extracts represent the peptidic fractions obtained after ultrafiltration (<3 kDa) of water-soluble peptides extracted from PR samples. PR12: Parmigiano-Reggiano at 12 months of ripening. PR18: Parmigiano-Reggiano at 18 months of ripening. PR24: Parmigiano-Reggiano at 24 months of ripening. PR30: Parmigiano-Reggiano at 30 months of ripening. Results were expressed as IC_50_, namely the peptides concentration (expressed in mg/mL) able to inhibit the enzymatic activity or the advanced glycation end-product formation by 50%. The IC_50_ values were calculated by plotting the peptide concentration (base-10 logarithm) as a function of the percentage of inhibition. Values are means of three independent digestions ± standard deviation (SD). Different letters indicate significantly different values (*p* < 0.05).

As reported in Figure 2A,B, water-soluble low-molecular weight peptides extracted from PR samples at different ripening stages were able to inhibit the activity of both α-amylase and α-glucosidase, with IC_50_ values ranging from 1.90 and 2.60 mg/mL for α-amylase, and between 2.74 and 3.50 mg/mL for α-glucosidase. In general, the peptide extracts were more effective in inhibiting α-amylase in respect to α-glucosidase at each ripening time. For both the enzymes, there is a clear evidence of ripening time-inhibitory activity relation, with some differences. In the case of α-amylase, the inhibitory activity decreased as the ripening time increased, with the PR12 sample being the most effective and PR30 the lowest (Figure 2A). Even in the case of α-glucosidase, the PR12 sample was found to be the strongest inhibitor (Figure 2B). The inhibitory potency decreased as the ripening time increased, with the PR24 and PR30 samples showing the lowest activity. Some studies have already highlighted the ability of peptides extracted from cheeses (Prato cheese, Minas Frescal cheese, Himalayan cheese, and Akawi cheese) to inhibit α-amylase and α-glucosidase; however, the lack of IC_50_ values makes an across-the-board comparison between the results difficult [19,37,38]. Confronting previously reported data, PR peptide extracts were less active against α-amylase in respect to a red seaweed hydrolysate (IC_50_ = 0.9 mg/mL) and a camel milk protein hydrolysate (IC_50_ = 0.03 mg/mL). The higher PR peptide extracts’ effectiveness was ascribed to the inhibition of α-glucosidase when compared, for instance, to a protein hydrolysate of sprouted quinoa yogurt beverage (IC_50_ = 8.86 mg/mL) and soy protein hydrolysate (IC_50_ = 4.94 mg/mL) [39,40].

Like the previous enzymes, DPP-IV-inhibitory activity decreased as the ripening time increased and was higher in PR12 and PR18 samples respect to PR24 and PR30 (Figure 2C). No significant differences were observed between PR12 and PR18 samples (*p* > 0.05) and between PR24 and PR30 samples (*p* > 0.05). Uenishi et al. [41] found that the DPP-IV inhibitory activity increased during ripening (from 0 to 12 months) of Gouda cheese, albeit they did not report the IC_50_ values. Moreover, Solieri et al. [21] found considerable differences among samples when the DPP-IV inhibitory activity was tested from 12-months ripening PR peptide extracts.

Considering the liquid secretions in the different gut compartments, as suggested by Minekus et al. [42], a solid meal should reach the small intestine diluted about 8 to 10 times. As an example, it is possible to estimate the amount of cheese needed to be consumed to have a significant effect on enzyme activities, considering the peptide concentration in the PR12 sample (63.54 mg/g of cheese), the cheese dilution factor in the small intestine (10 times), and the reported IC_50_ values for the three different enzymes (Figure 1). In the case of PR12 cheese, ingested amounts of 15, 22, and 6 g should be enough to reach peptide concentrations in the small intestine comparable to the IC_50_ values for α-amylase, α-glucosidase, and DPP-IV, respectively. Therefore, a serving size of cheese (50 g) could supply enough peptides to completely inhibit the activity of the enzymes.

Figure 2D reported the IC_50_ values of the water-soluble low-molecular weight peptide extracts from PR at different ripening times against fluorescent AGEs formation. As observed, PR12 and PR18 samples were more efficient inhibitors of fluorescent AGEs formation (IC_50_ = 6.35 and 6.46 mg/mL, respectively) respect to the PR24 and PR30 samples (IC_50_ = 8.17 and 7.67 mg/mL, respectively).

Some peptides have been previously exploited as AGE inhibitors. In particular, the tri-peptide glutathione and some γ-glutamylcysteine derivatives from aged garlic were effective AGE inhibitors [43,44]. Additional di-peptides containing tryptophan (NW and QW), as well as di-peptides containing histidine (carnosine, homocarnosine, and anserine) were able to inhibit protein glycation [44,45]. It has been speculated that the antioxidant properties of these peptides may be pivotal for the inhibitory activity [45].

### 3.2. Peptidomics Profiles of Water-Soluble Low-Molecular Weight Peptide Extracts of Parmigiano-Reggiano (PR) Samples

Peptidomics analysis allowed the identification of 415 unique peptides, considering all of the PR samples. The complete list of identified peptides together with the mass spectrometry data is reported in Table S1.

**Figure 3:**
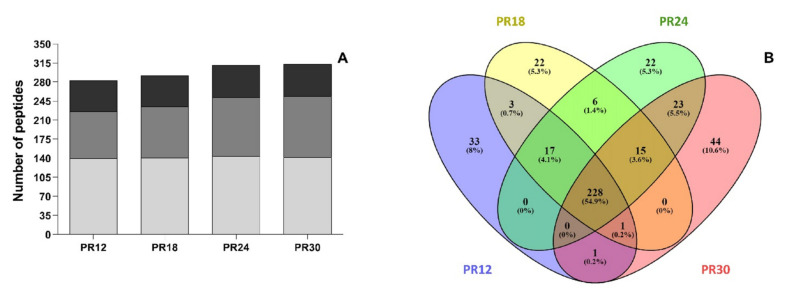
Number of peptides identified in the Parmigiano-Reggiano (PR) peptide fractions. (**A**) Number of peptides identified in the PR water-soluble peptide extracts at 12 (PR12), 18 (PR18), 24 (PR24), and 30 (PR30) months of ripening. The incidence of the different milk proteins on the released peptides is also shown. Gray identified the number of peptides released from β-casein. Dark gray identified the number of peptides released from αS1-casein. Black identified the number of peptides released from αS2-casein. (**B**) Venn diagram created with all the identified peptides in PR water-soluble peptide extracts at 12, 18, 24, and 30 months of ripening (see online Supplementary Material Table S1 for the peptide sequences).

The number of peptides in each PR sample slightly increased as a function of ripening time from 283 in PR12 to 312 in PR30 samples (Figure 3A) in accordance with previous studies [26,46]. In each sample, most of the identified peptides derived from β-casein followed by αS1-casein and αS2-casein. However, the percentage incidence changed during ripening. Even if the total number of β-casein-derived peptides remained approximately constant during ripening (140, 141, 144, and 142 peptides in PR12, PR18, PR24, and PR30 samples, respectively), the percentage incidence decreased as a function of the ripening time (49.5%, 48.3%, 46.5%, and 45.5% in PR12, PR18, PR24, and PR30 samples, respectively). Similarly, the percentage incidence of αS2-casein-derived peptides decreased over time passing from 20.1% (PR12) to 18.9% (PR30). On the contrary, both the number and the percentage incidence of αS1-casein-derived peptides increased as the ripening time increased. The number of αS1-casein-derived peptides and the percentage incidence grew from 86% and 30.4% in PR12 to 112% and 35.6% in PR30, respectively.

A Venn diagram (Figure 3B) revealed that 228 peptides (corresponding to the 54.9% of total peptides) were in common among the four PR samples. The highest number of unique peptides was found for the PR30 sample (44 unique peptides) followed by PR12 sample (33 unique peptides).

### 3.3. Identification of Bioactive Peptides in Comparison with Databases

The list of the peptides identified in the water-soluble low-molecular weight peptide extracts was compared for sequence matches with previously reported bioactive peptides, by using BIOPEP and MBPDB databases. A total of 49 peptides showed sequence homology (100%) with known bioactive peptides found in databases (Table 1). In accordance with Martini et al. [26], the number of bioactive peptides increased according to the ripening from 30, 35, and 41 peptides in PR12, PR18, and PR24 samples, respectively. Afterwards, a slight decline in the number of bioactive peptides was observed in PR30 sample (39 identified bioactive peptides) probably because of the extensive hydrolysis made by proteases and cytoplasmic peptidases released in cheese during lactic acid bacteria cell lysis [18]. Regardless, most bioactive peptides (26 peptides corresponding to the 54.2% of identified bioactive peptides) were in common between the four PR samples. A total of 13 bioactive peptides have been already detected in PR at different ripening stages [21,26], while 36 were identified for the first time in this work.

Among the 49 identified bioactive peptides, the majority (35 peptides) have been previously characterized as ACE-inhibitors. The tripeptides VPP and IPP have been already detected and quantified in PR samples at different ripening stages [21,25,26]. They received particular attention in the last years since they showed very low IC_50_ values against ACE and exhibited in vivo an anti-hypertensive effect on mild hypertensive subjects [47]. The β-casein-derived peptides LHLPLP and LPLP displayed low IC_50_ values against ACE and were found to be powerful decreasing agents of systolic blood pressure in spontaneously hypertensive rats [48,49].

The second most abundant class of identified bioactive peptides were anti-microbial peptides (7 identified peptides). The αS1-casein-derived anti-microbial peptide isracidin, corresponding to the sequence RPKHPIKHQGLPQEVLNENLLRF, has already confirmed an in vivo effect, protecting mice from *Staphylococcus aureus*, *Streptococcus pyogenes*, and *Listeria monocytogenes* infections [50].

Additional identified peptides belonged to the class of antioxidant (6 identified peptides), immunomodulatory (5 identified peptides), and anti-inflammatory (4 identified peptides) peptides, respectively (Table 1).

Despite the inhibitory effect of PR water-soluble low-molecular weight peptide extracts on DPP-IV activity, only 4 peptides with previously reported inhibitory activity were detected (Table 1) [41,51,52]. Among them, LPQ was the strongest DPP-IV inhibitor (IC_50_ = 82 μmol/L) and was detected in PR18, PR24, and PR30 samples. Moreover, peptides WIQP (IC_50_ = 237 μmol/L) and IPPLTQTPV (IC_50_ = 465 μmol/L) were identified only in PR24 sample and PR18 and PR24 samples, respectively. Peptide IPPL (IC_50_ = 429 μmol/L) was the only one identified in all the four PR samples.

Hence, considering the four above-mentioned peptides, their samples’ occurrence, and the lower IC_50_ values for PR12 and PR18 samples, a connection between the identified DPP-IV inhibitory peptides and the in vitro assessed DPP-IV inhibitory activity could not be stated.

Concerning α-glucosidase, only the peptide PFP (IC_50_ = 8.6 mmol/L), with previously reported activity [53], was detected in all four PR samples. Finally, no α-amylase inhibitory peptides were identified in the PR samples.

Overall, these data suggested the presence of new DPP-IV, α-glucosidase, and α-amylase inhibitory peptides in PR samples.

### 3.4. Selection of Potential Anti-Diabetic Peptides by In Silico Approach and Structure–Activity Relationship Modeling

To identify potential new inhibitory peptides inside the pool of peptides scored in PR samples through peptidomics technique, an approach that combined in silico analysis and structure activity relationship modeling was applied. Firstly, the list of peptides, after deletion of post-translational modified peptides, was screened by using PeptideRanker software. This software is a general predictor of peptide bioactivity, which allows for a quick and fast screening of hundreds of peptides, providing a score of increasing probability between 0 and 1 [35]. As suggested by the authors we selected a threshold of 0.5, which means that all the peptides with score between 0.5 and 1 were considered as potential bioactive. As reported in Supplementary Table S2, among the 263 peptides tested, 83 peptides had a score higher than 0.5 and were, therefore, selected for further analysis. Then, the potential bioactive peptides were explored by using PepSite 2, which is able to predict the binding probability (*p*-value) of a given peptide with a specific binding site (i.e., enzymes active sites) [36]. Among the 83 peptides selected by PeptideRanker, 13 peptides were excluded since the maximum permitted peptide length for PepSite 2 analysis is 10 amino acid residues. By setting a *p*-value cut-off of 0.01, we found 53, 56 and 51 peptides, which significantly interact with DPP-IV, α-glucosidase and α-amylase active sites, respectively (Tables S3–S5).

A subsequent analysis has been carried out by structure–activity relationship modeling approach, to identify with higher confidence potential inhibitory peptides for the next in vitro analysis. Previous studies evidenced that DPP-IV-inhibitory peptides possess typical structural characteristics. The sequence consensus of DPP-IV inhibitory peptides includes a branch-chained amino acid (I, L, A, M, F and W) at the N-terminus and a P or A residue in second position [54,55,56]. As reported in Table S3, 7 of the potential DPP-IV-inhibitory peptides screened by PeptideRanker and PepSite 2 had both these features. One peptide, IPPL, was already characterized as a DPP-IV inhibitor and, therefore, no further considered [52]. Since DPP-IV is a membrane-bound enzyme located in the brush-border of intestinal enterocytes, only peptides that are not hydrolyzed by digestive proteases may rationally reach intact the biological target (i.e., DPP-IV) and may possibly be active in vivo. Therefore, the 6 potential DPP-IV-inhibitory peptides were subjected to in silico gastro-intestinal digestion to check their stability to gastro-intestinal proteases. As reported in Table S3, peptides FALPQ, IPPLT and LPLP were unstable under gastro-intestinal conditions, whereas the remaining peptides (IPP, LPPT, and APFPE) were selected as the best candidates for further in vitro validation.

The structural properties of α-glucosidase inhibitory peptides have been recently described [57]. The most potent α-glucosidase inhibitory peptides show a hydroxyl group-containing (S, T or Y) or a basic (K or R) amino acid residue at the N-terminus, as well as a hydrophobic amino acid (M, A or F) at the C-terminus. Moreover, the presence of a P residue close to the C-terminal end appeared to be of paramount importance for the inhibitory activity [57]. As reported in Table S4, 7 potential bioactive peptides possessed at least two of these features. Among them, peptides KIHPF, IHPF, DKIHPF and EPF were found to be cleaved by gastro-intestinal proteases. Accordingly, the stable peptides PPF, VVPPF and VVVPPF were selected for in vitro analysis and validation.

Unfortunately, no clear structure–activity relationship has been found in literature for α-amylase-inhibitory peptides and no further peptides were selected for this inhibitory activity.

### 3.5. In Vitro Inhibitory Activity of Synthetic Selected Peptides

Synthetic selected peptides were then tested for their ability to inhibit the enzymes DPP-IV, α-glucosidase, and α-amylase (Table 2).

Among the three peptides that shared structural characteristics of DPP-IV inhibitors, APFPE was found to be the most potent inhibitor followed by IPP and LPPT. Peptide APFPE showed IC_50_ value of 49.5 ± 0.5 μmol/L, like the most potent DPP-IV inhibitory peptides identified in milk proteins [54,55,56,57]. The tripeptide IPP was found to be 3.4 times less potent than APFPE. This tripeptide shared the N-terminal sequence IP with previously identified DPP-IV inhibitory tripeptides such as IPI, IPA, and IPM [52]. IPP displayed similar inhibitory potency as the dipeptide IP and was 2.5 more effective than IPPL [52]. The lactotripeptides IPP is a multifunctional bioactive peptide with previously reported ACE-inhibitory, antioxidant, and anti-inflammatory activities [58]. It has been detected in fermented milk, yoghurt, cheeses, and after digestion of several types of milk and cheeses [18,26].

The tripeptide IPP gained peculiar attention in the last years due to the reported in vivo anti-hypertensive effect in human [58]. The tetrapeptide LPPT also showed in vitro DPP-IV inhibitory activity with IC_50_ of the same magnitude order of the parent tripeptide LPP [52]. Moreover, the tripeptide PPF, previously selected for its potential α-glucosidase inhibitory activity, also showed relatively high DPP-IV inhibitory activity (Table 2). This peptide is characterized by the presence of a P residue at the second position, typical of most DPP-IV inhibitory peptide. The longer peptides, VVPPF and VVVPPF, showed a 6 times lower inhibitory activity in respect to PPF, suggesting that the addition of V residues at the N-terminal negatively affects the peptide activity.

To support our findings, HPEPDOCK Server was used to create the binding models and to predict the possible interaction between the selected peptides (IPP and APFPE) and the DPP-IV binding sites. In fact, the Supplementary Figure S1 pointed out the predicted blind peptide–protein docking by modeling of peptides conformations and structures suggesting a possible interaction between the peptides and the DPP-IV active site.

Concerning the peptides selected for their potential α-glucosidase inhibitory effect (PPF, VVPPF, and VVVPPF), they failed to show appreciable inhibitory activity with IC_50_ values above the maximum tested concentration of 1 mmol/L. At this concentration, percentages of inhibition of 22.5 ± 1.0%, 22.0 ± 1.3%, and 8.8 ± 1.3 were observed for VVVPPF, VVPPF, and PPF, respectively. Surprisingly, IPP resulted the most active peptide against α-glucosidase activity, even if it was not selected as potential inhibitor of this enzyme.

Previous studies revealed that α-glucosidase inhibitors might also act as inhibitory agents against α-amylase enzymatic activity [59]. Therefore, the same peptides were tested against α-amylase activity. As reported in Table 2, only IPP exerted a weak inhibitory activity against α-amylase, whereas the other peptides were ineffective at the maximum tested concentration of 1 mmol/L. These latter results suggest the need of more detailed and robust studies on the structure–activity relationship of potential bioactive peptides and α-glucosidase and α-amylase inhibitory effect for the correct prediction of novel inhibitory peptides.

## 4. Conclusions

In the present study, the potential anti-diabetic activity of PR cheese was demonstrated over ripening time for the first time, strengthening the functional and healthy role of this cheese in human nutrition. The peptide fractions extracted from PR cheese exhibited DPP-IV, α-glucosidase, α-amylase inhibitions, and fluorescent AGE-inhibitory activities, which generally decreased as a function of ripening time. Several previously identified bioactive peptides were detected in PR peptide fractions, whose number increased as a function of ripening time.

Furthermore, a cheap and fast method was presented for the selection of potential new bioactive peptides from chemical complex hydrolysates. This approach combined peptidomics analysis with in vitro biological activity assays, in silico analysis, and structure–activity relationship modeling. The application of this method allowed for the identification of a new potent DPP-IV inhibitory peptide (APFPE) in PR cheese and made possible the identification of new biological activities (i.e., inhibition of target enzymes for diabetes) for the multifunctional peptide IPP. Therefore, the novelty of this study lies in the development of a new, quick, and cost-effective integrated methodology supported by confirmed in vitro evidences. However, results suggested that the proposed method works well when robust structure–activity relationship studies are available in literature (i.e., DPP-IV). This was evident when the selected peptides were tested for in vitro α-glucosidase inhibitory activity. The candidates chosen in the light of the available structure–activity relationship studies on α-glucosidase failed to show substantial activity, whereas the most potent peptide was IPP, which was not selected with this approach. Therefore, the fully application of the proposed method underlined the necessity of in-depth structure–activity relationship studies that make the in vitro selection of novel bioactive peptide candidates for subsequent in vivo validation more predictable. This is the most important limit related to the application of the proposed combined approach. However, the described procedure has several advantages due to its cheapness, quickness, and less laborious analytics in respect to the classical conventional approach used in bioactive peptide discovery, overcoming the lack of physiological significance of pure in silico approaches. Indeed, this method can be theoretically applied to any type of food hydrolysate, simplifying the identification process of novel bioactive peptides.

## Figures and Tables

**Table 1:** Peptides with previously reported biological activity identified in water-soluble low-molecular weight peptide extracts from Parmigiano-Reggiano (PR) at different ripening times.

Peptide Sequence	Protein Precursor	Sample	Bioactivity ^a^
DKIHP	β-casein f(47–51)	PR24	ACE-inhibition
DKIHPF	β-casein f(47–52)	PR12, PR18, PR24, PR30	ACE-inhibition
DVPSERYLG	αS1-casein f(85–93)	PR30	ACE-inhibition
EMPFPK	β-casein f(108–113)	PR24, PR30	ACE-inhibition Anti-microbial
ENLLRF	αS1-casein f(18–23)	PR12, PR18, PR24, PR30	ACE-inhibition
FFVAP	αS1-casein f(23–27)	PR24	ACE-inhibition
FGK	αS1-casein f(32–34)	PR12, PR18, PR24, PR30	ACE-inhibition
FVAP	αS1-casein f(24–27)	PR12, PR18, PR24, PR30	ACE-inhibition
GTQY	αS1-casein f(170–172)	PR18, PR24, PR30	ACE-inhibition Antioxidant
IPP	β-casein f(74–76)	PR12, PR18, PR24, PR30	ACE-inhibition Antioxidant Anti-inflammatory
IPPL	β-casein f(74–77)	PR12, PR18, PR24, PR30	DPP-IV-inhibition
IPPLTQTPV	β-casein f(74–82)	PR18, PR24	DPP-IV-inhibition
IVP	αS1-casein f(71–73)	PR12, PR18, PR24, PR30	ACE-inhibition
LEE	β-casein f(3–5)	PR12, PR18, PR24, PR30	ACE-inhibition
LHLPLP	β-casein f(133–138)	PR12, PR18	ACE-inhibition
LLY	β-casein f(191–193)	PR18	Antioxidant Immunomodulation Anti-inflammatory
LNF	αS2-casein f(161–163)	PR12, PR18, PR24, PR30	ACE-inhibition
LPLP	β-casein f(135–138)	PR12, PR18, PR24	ACE-inhibition
LPQ	β-casein f(70–72) αS1-casein f(11–13) αS2-casein f(176–178)	PR18, PR24, PR30	DPP-IV-inhibition
LVYPFP	β-casein f(58–63)	PR12, PR18, PR24, PR30	ACE-inhibition
LVYPFPGP	β-casein f(58–65)	PR12, PR18, PR24, PR30	ACE-inhibition
PEL	αS1-casein f(147–149)	PR12, PR18, PR24, PR30	Antioxidant
PFP	β-casein f(61–63)	PR12, PR18, PR24, PR30	ACE-inhibition α-glucosidase inhibition
PFPE	αS1-casein f(27–30)	PR12, PR18, PR24, PR30	ACE-inhibition
PGPIP	β-casein f(63–67)	PR12, PR18, PR24, PR30	ACE-inhibition
PGPIPN	β-casein f(63–68)	PR12, PR18, PR24, PR30	ACE-inhibition Immunomodulation Anti-inflammatory
PLW	αS1-casein f(197–199)	PR30	ACE-inhibition
QEPV	β-casein f(194–197)	PR12, PR18, PR24, PR30	Immunomodulation
QGP	αS2-casein f(101–103)	PR18, PR24	ACE-inhibition
QGPIVLNPWDQVKR	αS2-casein f(101–114)	PR30	Antioxidant
RELEEL	β-casein f(1–6)	PR12, PR18, PR24, PR30	Antioxidant
RPKHPIKHQGLPQEVLNENLLRF	αS1-casein f(1–23)	PR30	Immunomodulation Anti-microbial
SLPQ	β-casein f(69–72)	PR12, PR18, PR24, PR30	ACE-inhibition
TEDELQDKIHPF	β-casein f(41–52)	PR24, PR30	Anti-microbial
TKVIPYVRYL	αS2-casein f(198–207)	PR18, PR24, PR30	Anti-microbial
TVY	αS2-casein f(182–184)	PR12, PR18, PR24, PR30	ACE-inhibition
VEP	β-casein f(116–118)	PR24, PR30	ACE-inhibition
VLP	β-casein f(170–172)	PR12, PR18, PR24, PR30	ACE-inhibition
VPP	β-casein f(84–86)	PR12, PR18, PR24, PR30	ACE-inhibition Antioxidant Anti-inflammatory
VVPP	β-casein f(83–86)	PR12, PR18, PR24, PR30	ACE-inhibition
VVVPPF	β-casein f(82–87)	PR12, PR18, PR24, PR30	ACE-inhibition
WIQP	αS2-casein f(193–196)	PR24	ACE-inhibition DPP-IV-inhibition
YLEQLLR	αS1-casein f(94–100)	PR24, PR30	Anti-microbial
YLG	αS1-casein f(91–93)	PR12, PR18, PR24, PR30	Antioxidant
YLGY	αS1-casein f(91–94)	PR12	ACE-inhibition Antioxidant
YQEP	β-casein f(193–196)	PR12	ACE-inhibition
YQEPVLGPVRGPFPIIV	β-casein f(193–209)	PR24, PR30	ACE-inhibition Anti-microbial
YQGPIVLNPWDQVKR	αS2-casein f(100–114)	PR12, PR18, PR24, PR30	ACE-inhibition Anti-microbial Immunomodulation
YQL	αS1-casein f(154–156)	PR18, PR24, PR30	Antioxidant

^a^ Potential bioactivities were achieved from the BIOPEP database and the Milk Bioactive Peptides Database. ACE: angiotensin converting enzyme; DPP-IV: dipeptidyl peptidase IV.

**Table 2:** Inhibitory activity of synthetic selected peptides against dipeptidyl peptidase IV (DPP-IV), α-glucosidase, and α-amylase; results are expressed as IC_50_^a^.

Peptide Sequence	DPP-IV μmol/L	α-Glucosidase μmol/L	α-Amylase μmol/L
APFPE	49.5 ± 0.5	n.a.	n.a.
IPP	168.8 ± 2.2	764.5 ± 15.0	763.5 ± 18.9
LPPT	495.3 ± 3.6	n.a.	n.a.
PPF	141.9 ± 1.7	>1000	n.a.
VVPPF	698.7 ± 11.1	>1000	n.a.
VVVPPF	609.7 ± 13.6	>1000	n.a.

^a^ IC_50_ is defined as the concentration of peptide, expressed in μmol/L, able to inhibit the enzymatic activity by 50%. n.a. means that the peptide was not active against the specific enzyme.

## Data Availability

All the data was shown in the article and in Supplementary Materials.

## Supplementary Materials

The following are available online at https://www.mdpi.com/article/10.3390/biology10060563/s1, Table S1: List of peptides identified in the Parmigiano-Reggiano samples after 12 (PR12), 18 (PR18), 24 (PR24), and 30 (PR30) months of ripening, Table S2: Potential bioactive peptides (threshold > 0.5) identified though PeptideRanker software, Table S3: Selection of potential DPPIV-inhibitory peptides as a function of PepSite *P*-value, structure–activity relationship (SAR) modeling, and in silico gastro-intestinal digestion (GID) stability, Table S4: Selection of potential α-glucosidase-inhibitory peptides on the basis on the PepSite *P*-value, structure–activity relationship (SAR) modeling, and in silico gastro-intestinal digestion (GID) stability, Table S5: PepSite *P*-value for α-amylase, Figure S1: Peptides binding site prediction of DPP-IV enzyme with IPP and APFPE.
