# Supplementary material for: An Integrated Peptidomics and In Silico Approach to Identify Novel Anti-Diabetic Peptides in Parmigiano-Reggiano Cheese
Source: Biology (Basel). 2021 Jun 21;10(6):563. doi: 10.3390/biology10060563 (PMC8234620; doi:10.3390/biology10060563)
Supplement: Supplementary file 1 [file biology-10-00563-s001.zip › Figure S1.pdf]

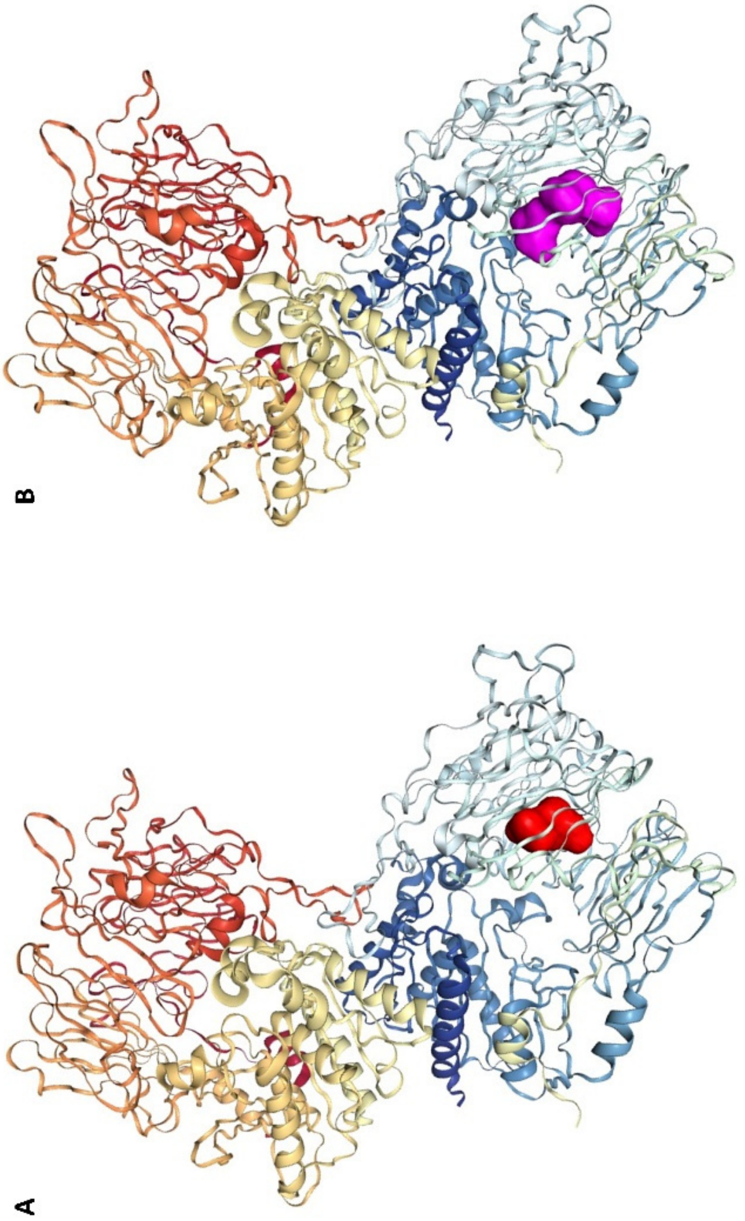

Figure S1. Peptides binding site prediction of DPP-IV enzyme (PDB ID entry code: 1NU6) with IPP (A) and APFPE (B).
