# Supplementary material for: An Integrated Peptidomics and In Silico Approach to Identify Novel Anti-Diabetic Peptides in Parmigiano-Reggiano Cheese
Source: Biology (Basel). 2021 Jun 21;10(6):563. doi: 10.3390/biology10060563 (PMC8234620; doi:10.3390/biology10060563)
Supplement: Supplementary file 1 [file biology-10-00563-s001.zip › Table S1.pdf]

Table S1. List of peptides identified in the Parmigiano Reggiano samples after 12 (PR12), 18 (PR18), 24 (PR24) and 30 (PR30) months of ripening.

Start	End	Observed (*m*/*z*)	Calculated (MH^{+n})	Error (ppm)	Sequence	z	PR 12	PR 18	PR 24	PR 30
					β-casein-derived peptides					
1	5	338.1688	338.1690	-0.59	REELE	2	X	X	X	X
1	3	417.2445	417.2456	-2.64	REL	1			X	
1	4	546.2870	546.2882	-2.20	RELE	1	X	X	X	X
1	6	788.4135	788.4149	-1.78	RELEEL	1	X	X	X	X
1	11	642.8250	642.8251	-0.16	RELEELNVPGE	2	X			
2	6	632.3135	632.3137	-0.32	ELEEL	1	X	X	X	X
3	5	390.1869	390.1871	-0.51	LEE	1	X	X	X	X
3	6	503.2717	503.2712	0.99	LEEL	1	X	X	X	
4	6	390.1869	390.1871	-0.51	EEL	1	X	X	X	X
5	7	375.1870	375.1874	-1.07	ELN	1	X	X	X	X
6	10	499.2874	499.2875	-0.20	LNVPG	1				X
7	11	515.2452	515.2460	-1.55	NVPGE	1	X	X		
11	13	360.2128	360.2129	-0.28	EIV	1	X	X	X	X
11	14	489.2549	489.2555	-1.23	EIVE	1	X	X	X	X
12	14	360.2125	360.2129	-1.11	IVE	1	X	X	X	X
12	16	640.2959	640.2953	0.94	IVE**S**L	1	X	X	X	X
12	28	737.9606	737.9590	2.17	IVE**S**L**SSS**EESITRINK	3	X	X	X	X
12	25	928.8220	928.8239	-2.05	IVE**S**L**SSS**EESITR	2	X			
12	27	1042.3902	1042.3874	2.69	IVE**S**L**SSS**EESITRIN	2	X	X	X	X
13	15	334.1605	334.1609	-1.20	VES	1	X	X	X	X
13	16	527.2111	527.2113	-0.38	VE**S**L	1	X			
13	25	872.2811	872.2818	-0.80	VE**S**L**SSS**EESITR	2	X	X	X	X
13	27	657.5674	657.5660	2.13	VE**S**L**SSS**EESITRIN	3	X	X	X	X
13	28	700.2649	700.2643	0.86	VE**S**L**SSS**EESITRINK	3	X	X	X	X
14	28	667.2420	667.2415	0.75	E**S**L**SSS**EESITRINK	3	X	X	X	X
14	25	822.7476	822.7476	0.00	E**S**L**SSS**EESITR	2	X	X	X	X
14	27	936.3120	936.3111	0.96	E**S**L**SSS**EESITRIN	2	X	X	X	X
15	22	573.1081	573.1099	-3.14	**S**L**SSS**EES	2	X	X	X	X
15	22	1065.2432	1065.2462	-2.82	SL**SSS**EES	1	X	X	X	X
15	24	640.1928	640.1926	0.31	SL**SSS**EESIT	2	X	X	X	X
15	24	680.1741	680.1758	-2.50	**S**L**SSS**EESIT	2		X		
15	25	678.2600	678.2600	0.00	SLS**SS**EESITR	2		X		
15	25	718.2423	718.2432	-1.25	SL**SSS**EESITR	2	X	X	X	X
15	25	758.2261	758.2263	-0.26	**S**L**SSS**EESITR	2	X	X	X	X

15	26	774.7873	774.7852	2.71	SLSSSEESITRI	2	X	X	X	X
15	26	814.7691	814.7684	0.86	SLSSSEESITRI	2		X		
15	27	831.8084	831.8067	2.04	SLSSSEESITRIN	2	X	X	X	X
15	27	871.7898	871.7898	0.00	SLSSSEESITRIN	2	X	X	X	X
15	28	597.5728	597.5719	1.51	SLSSSEESITRINK	3	X	X	X	X
16	18	386.1306	386.1323	-4.40	LSS	1	X	X	X	X
16	18	466.0979	466.0986	-1.50	LSS	1	X	X	X	X
16	21	891.1786	891.1822	-4.04	LSSSEE	1	X	X	X	X
16	22	978.2115	978.2142	-2.76	LSSSEES	1	X	X	X	X
16	23	546.1528	546.1528	0.00	LSSSEESI	2	X	X	X	X
16	24	556.6930	556.6934	-0.72	LSSSEESIT	2	X			
16	24	596.6767	596.6766	0.17	LSSSEESIT	2	X	X	X	X
16	25	634.7437	634.7440	-0.47	LSSSEESITR	2	X	X	X	X
16	25	634.7438	634.7440	-0.32	LSSSEESITR	2	X	X	X	X
16	25	674.7270	674.7272	-0.30	LSSSEESITR	2	X	X	X	X
16	26	731.2708	731.2692	2.19	LSSSEESITRI	2	X	X	X	X
16	27	788.2888	788.2907	-2.41	LSSSEESITRIN	2	X	X	X	X
16	28	568.5627	568.5612	2.64	LSSSEESITRINK	3	X	X	X	X
16	28	595.2157	595.2166	-1.51	LSSSEESITRINK	3	X	X	X	X
16	28	541.9043	541.9057	-2.58	LSSSEESITRINK	3	X	X	X	X
17	22	778.0945	778.0981	-4.63	SSSEE	1		X		
17	22	865.1258	865.1301	-4.97	SSSEES	1	X	X	X	X
17	24	1079.2592	1079.2619	-2.50	SSSEESIT	1	X	X	X	X
17	25	578.2012	578.2020	-1.38	SSSEESITR	2	X	X	X	X
17	25	618.1833	618.1851	-2.91	SSSEESITR	2	X	X	X	X
17	26	674.7286	674.7272	2.07	SSSEESITRI	2	X	X	X	X
17	27	691.7600	691.7655	-7.95	SSSEESITRIN	2	X	X	X	X
17	27	731.7470	731.7486	-2.19	SSSEESITRIN	2	X	X	X	X
17	28	755.8103	755.8129	-3.44	SSSEESITRINK	2	X	X	X	X
17	28	795.7949	795.7961	-1.51	SSSEESITRINK	2	X	X	X	X
17	31	654.2753	654.2737	2.45	SSSEESITRINKKIE	3	X			
18	21	611.0975	611.0997	-3.60	SSEE	1	X	X	X	X
18	22	618.1625	618.1654	-4.69	SSEES	1		X	X	
18	27	648.2490	648.2494	-0.62	SSEESITRIN	2	X	X	X	X
18	22	698.1291	698.1318	-3.87	SSEES	1	X	X	X	X
18	24	912.2607	912.2635	-3.07	SSEESIT	1	X	X	X	X
18	25	988.3946	988.3983	-3.74	SSEESITR	1	X	X	X	X
18	25	1068.3604	1068.3646	-3.93	SSEESITR	1	X	X	X	X
18	25	574.6683	574.6691	-1.39	SSEESITR	2	X			
18	28	448.5444	448.5449	-1.11	SSEESITRINK	3	X			

18	28	475.2006	475.2004	0.42	**SS**EESITRINK	3	X	X	X	X
19	25	451.1866	451.1868	-0.44	**S**EESITR	2	X	X	X	X
20	23	557.1842	557.1855	-2.33	EE**S**I	1	X	X	X	
20	25	367.6874	367.6876	-0.54	EESITR	2	X	X	X	X
21	23	348.1762	348.1765	-0.86	ESI	1	X	X	X	X
23	25	389.2491	389.2507	-4.11	ITR	1	X			
29	31	389.2377	389.2395	-4.62	KIE	1	X	X	X	X
31	33	423.2231	423.2238	-1.65	EKF	1		X	X	X
32	34	422.2380	422.2398	-4.26	KFQ	1		X		
32	37	424.1645	424.1653	-1.89	KFQ**S**EE	2	X	X	X	X
32	39	552.2231	552.2239	-1.45	KFQ**S**EEQQ	2	X	X	X	X
32	40	616.2525	616.2532	-1.14	KFQ**S**EEQQQ	2	X	X	X	X
32	42	731.2982	731.2983	-0.14	KFQ**S**EEQQQTE	2	X	X	X	X
32	43	788.8101	788.8118	-2.16	KFQ**S**EEQQQTED	2	X	X	X	X
32	44	649.6053	649.6054	-0.15	KFQ**S**EEQQQTEDELQ	3	X	X	X	X
33	36	590.1851	590.1858	-1.19	FQ**S**E	1	X	X	X	X
33	37	719.2257	719.2284	-3.75	FQ**S**EE	1	X	X	X	X
33	39	488.1752	488.1764	-2.46	FQ**S**EEQQ	2	X	X	X	X
33	43	667.2497	667.2508	-1.65	FQ**S**EEQQQTE	2	X	X	X	X
34	39	828.2733	828.2771	-4.59	Q**S**EEQQ	1		X		
38	46	559.7505	559.7516	-1.97	QQQTEDELQ	2				X
40	48	553.2529	553.2540	-1.99	QTEDELQDK	2	X	X	X	X
41	46	734.3191	734.3203	-1.63	TEDELQ	1	X	X	X	X
41	48	489.2243	489.2247	-0.82	TEDELQDK	2				X
41	52	491.2415	491.2403	2.44	TEDELQDKIHPF	3			X	X
42	46	633.2711	633.2726	-2.37	EDELQ	1	X	X	X	X
43	46	504.2291	504.2300	-1.78	DELQ	1	X	X	X	X
43	47	619.2554	619.2570	-2.58	DELQD	1	X	X	X	
43	52	621.3129	621.3117	1.93	DELQDKIHPF	2				X
44	46	389.2027	389.2031	-1.03	ELQ	1	X	X	X	X
45	52	499.2775	499.2769	1.20	LQDKIHPF	2				X
46	48	390.1968	390.1983	-3.84	QDK	1		X		
47	51	305.1706	305.1714	-2.62	DKIHP	2			X	
47	52	378.7052	378.7056	-1.06	DKIHPF	2	X	X	X	X
48	52	321.1921	321.1921	0.00	KIHPF	2	X	X	X	X
49	52	513.2818	513.2820	-0.39	IHPF	1	X	X	X	X
57	68	650.8517	650.8504	2.00	SLVYPFPGPIPN	2	X	X	X	X
58	63	735.4090	735.4076	1.90	LVYPFP	1	X	X	X	X
58	65	445.2455	445.2445	2.25	LVYPFPGP	2	X	X	X	X
58	68	607.3345	607.3344	0.16	LVYPFPGPIPN	2	X	X	X	X

61	63	360.1918	360.1918	0.00	PFP	1	X	X	X	X
63	65	383.2287	383.2289	-0.52	PGPI	1	X	X	X	X
63	67	480.2821	480.2817	0.83	PGPIP	1	X	X	X	X
63	68	594.3245	594.3246	-0.17	PGPIPN	1	X	X	X	X
64	68	497.2709	497.2718	-1.81	GPIPN	1			X	X
65	67	326.2074	326.2074	0.00	PIP	1	X	X	X	X
65	68	440.2502	440.2504	-0.45	PIPN	1	X	X	X	X
66	69	430.2297	430.2296	0.23	IPNS	1				X
68	72	558.2874	558.2882	-1.43	NSLPQ	1			X	
69	72	444.2450	444.2453	-0.68	SLPQ	1	X	X	X	X
70	72	357.2118	357.2132	-3.92	LPQ	1		X	X	X
73	77	553.3356	553.3344	2.17	NIPPL	1			X	
73	91	696.0588	696.0576	1.72	NIPPLTQTPVVVPPFLQPE	3				X
74	76	326.2074	326.2074	0.00	IPP	1	X	X	X	X
74	77	439.2922	439.2915	1.59	IPPL	1	X	X	X	X
74	78	540.3405	540.3392	2.41	IPPLT	1	X	X	X	X
74	82	483.2849	483.2869	-4.14	IPPLTQTPV	2		X	X	
74	91	658.0452	658.0433	2.89	IPPLTQTPVVVPPFLQPE	3	X			
77	79	361.2067	361.2082	-4.15	LTQ	1	X	X	X	X
79	81	444.2444	444.2453	-2.03	QTPV	1			X	X
79	88	548.8253	548.8237	2.92	QTPVVVPPFL	2	X	X	X	X
80	88	968.5525	968.5515	1.03	TPVVVPPFL	1	X	X	X	X
80	92	661.8729	661.8714	2.27	TPVVVPPFLQPE	2	X			
80	93	776.9323	776.9258	8.37	TPVVVPPFLQPEVM	2			X	
81	83	314.2076	314.2074	0.64	PVV	1				X
82	84	316.2229	316.2231	-0.63	VVV	1			X	
82	86	510.3285	510.3286	-0.20	VVVPP	1	X	X	X	X
82	87	329.2020	329.2022	-0.61	VVVPPF	2	X	X	X	X
82	90	498.2310	498.2299	2.21	VVVPPFLPQ	2	X			
82	91	562.8219	562.8211	1.42	VVVPPFLQPE	2			X	
82	93	677.8759	677.8756	0.44	VVVPPFLQPEVM	2				X
82	88	770.4805	770.4811	-0.78	VVVPPFL	1	X	X	X	X
83	86	411.2604	411.2602	0.49	VVPP	1	X	X	X	X
83	87	558.3289	558.3286	0.54	VVPPF	1	X	X	X	X
83	88	671.4140	671.4127	1.94	VVPPFL	1	X	X	X	X
84	86	312.1915	312.1918	-0.96	VPP	1	X	X	X	X
84	88	572.3448	572.3443	0.87	VPPFL	1			X	X
85	87	360.1917	360.1918	-0.28	PPF	1	X			
86	88	376.2241	376.2231	2.66	PFL	1	X	X	X	X
87	93	863.4355	863.4332	2.66	FLQPEVM	1				X

88	91	486.2555	486.2558	-0.62	LQPE	1	X	X	X	X
90	92	344.1816	344.1816	0.00	PEV	1		X	X	X
90	93	475.2236	475.2221	3.16	PEVM	1	X			
91	93	378.1688	378.1693	-1.32	EVM	1		X		
92	97	310.6745	310.6754	-2.90	VMGVSK	2		X		
92	98	368.207	368.2061	2.44	V**M**GVSKV	2	X			
100	102	350.1375	350.1380	-1.43	EAM	1	X	X	X	X
101	105	517.2797	517.2803	-1.16	AMAPK	1	X			
108	113	374.6879	374.6885	-1.60	EMPFPK	2			X	X
116	118	344.1814	344.1816	-0.58	VEP	1			X	X
116	119	491.2494	491.2500	-1.22	VEPF	1	X	X	X	X
117	119	392.1804	392.1816	-3.06	EPF	1				X
122	131	546.7746	546.7752	-1.10	SQSLTLTDVE	2	X			
129	131	362.1542	362.1558	-4.42	DVE	1	X	X	X	X
130	132	361.1705	361.1718	-3.60	VEN	1			X	
133	138	345.2218	345.2209	2.61	LHLPLP	2	X	X		
135	138	439.2923	439.2915	1.82	LPLP	1	X	X	X	
151	154	427.2554	427.2551	0.70	LPPT	1	X			
155	157	396.1957	396.1952	1.26	VMF	1			X	
163	166	499.2173	499.2164	1.80	LSL**S**	1				X
169	172	456.3174	456.3180	-1.31	KVLP	1	X	X	X	X
170	172	328.2232	328.2231	0.30	VLP	1	X	X	X	X
170	176	652.4015	652.4028	-1.99	VLPVPQ	1		X		
172	175	440.2503	440.2504	-0.23	PVPQ	1	X	X	X	X
191	193	408.2488	408.2493	-1.22	LLY	1		X		
193	196	536.2351	536.2351	0.00	YQEP	1	X			
193	209	941.0402	941.0353	5.21	YQEPVLGPVRGPFPIIV	2			X	X
194	196	373.1713	373.1718	-1.34	QEP	1		X	X	X
194	197	472.2400	472.2402	-0.42	QEPV	1	X	X	X	X
196	198	328.2229	328.2231	-0.61	PVL	1			X	X
199	209	576.3504	576.3504	0.00	GPVRGPFPIIV	2		X		
204	207	473.2761	473.2758	0.63	PFPI	1	X	X	X	X
206	208	342.2392	342.2387	1.46	PII	1	X	X	X	X
206	209	441.3069	441.3070	-0.23	PIIV	1				X
207	209	344.2544	344.2544	0.00	IIV	1	X	X	X	X
αS1-casein-derived peptides										
1	23	553.5170	553.5164	1.08	RPKHPIKHQGLPQEVLNENLLRF	5				X
10	12	414.2340	414.2347	-1.69	GLPQ	1		X		

14	18	603.2976	603.2984	-1.33	EVLNE	1		X		
17	19	379.7114	379.7111	0.79	NENLLR	2		X		
17	21	602.3144	602.3144	0.00	NENLL	1	X			
17	23	453.2471	453.2456	3.31	NENLLRF	2	X	X	X	X
18	23	396.2254	396.2241	3.28	ENLLRF	2	X	X	X	X
22	30	555.2926	555.2926	0.00	RFFVAPFPE	2		X		
23	25	412.2237	412.2231	1.46	FFV	1		X	X	X
23	27	580.3128	580.3130	-0.34	FFVAP	1			X	
24	27	433.2441	433.2445	-0.92	FVAP	1	X	X	X	X
24	29	677.3670	677.3657	1.92	FVAPFP	1	X	X	X	X
24	30	806.4076	806.4083	-0.87	FVAPFPE	1	X	X	X	X
24	32	526.7760	526.7762	-0.38	FVAPFPEVF	2				X
24	34	619.3358	619.3344	2.26	FVAPFPEVFGK	2	X	X	X	X
24	35	683.8562	683.8557	0.73	FVAPFPEVFGKE	2		X		
24	36	498.9391	498.9397	-1.20	FVAPFPEVFGKEK	3	X	X	X	X
24	37	569.9761	569.9750	1.93	FVAPFPEVFGKEKVN	3		X	X	X
24	41	542.0465	542.0465	0.00	FVAPFPEVFGKEKVNELSK	4				X
25	29	530.2986	530.2973	2.45	VAPFP	1	X	X	X	
25	30	659.3402	659.3399	0.46	VAPFPE	1	X	X	X	X
25	32	453.2429	453.2420	1.99	VAPFPEVF	2	X			
25	34	545.8019	545.8002	3.11	VAPFPEVFGK	2	X	X	X	X
26	30	560.2724	560.2715	1.61	APFPE	1	X			
27	30	489.2350	489.2344	1.23	PFPE	1	X	X	X	
30	32	394.1970	394.1973	-0.76	EVF	1	X	X	X	X
31	33	322.1757	322.1761	-1.24	VFG	1	X	X	X	X
31	34	450.2697	450.2711	-3.11	VFGK	1	X	X	X	X
32	34	351.2020	351.2027	-1.99	FGK	1	X	X	X	X
38	45	478.2097	478.2102	-1.05	NEL**S**KDIG	2	X			
41	47	408.1622	408.1628	-1.47	SKDIG**S**E	2				X
41	50	606.7070	606.7071	-0.16	SKDIG**S**E**S**TE	2			X	X
41	52	728.2496	728.2498	-0.27	SKDIG**S**E**S**TEDQ	2			X	X
41	54	829.2889	829.2886	0.36	SKDIG**S**E**S**TEDQAM	2				X
41	58	714.9456	714.9444	1.68	SKDIG**S**E**S**TEDQAMEDIK	3				X
42	50	563.1909	563.1911	-0.36	KDIG**S**E**S**TE	2	X	X	X	X
42	52	684.7324	684.7338	-2.04	KDIG**S**E**S**TEDQ	2		X	X	X
42	54	785.7725	785.7726	-0.13	KDIG**S**E**S**TEDQAM	2	X	X	X	X
42	55	850.2918	850.2939	-2.47	KDIG**S**E**S**TEDQAME	2	X	X	X	X
42	56	907.8072	907.8074	-0.22	KDIG**S**E**S**TEDQAMED	2				X
43	45	304.1497	304.1503	-1.97	DIG	1	X	X	X	X
43	46	391.1819	391.1823	-1.02	DIGS	1				X

43	54	721.7246	721.7251	-0.69	DIG**S**E**S**TEDQAM	2	X	X	X	X
44	46	356.1208	356.1217	-2.53	IG**S**	1	X			
44	54	664.2101	664.2117	-2.41	IG**S**E**S**TEDQAM	2			X	X
45	54	607.6693	607.6696	-0.49	G**S**E**S**TEDQAM	2	X	X	X	X
45	55	672.1900	672.1909	-1.34	G**S**E**S**TEDQAME	2	X	X	X	X
46	52	875.2634	875.2666	-3.66	SE**S**TEDQ	1			X	X
46	53	473.6548	473.6555	-1.48	SE**S**TEDQA	1				X
46	54	539.1752	539.1757	-0.93	SE**S**TEDQAM	2	X	X	X	X
46	54	579.1587	579.1589	-0.35	**S**E**S**TEDQAM	2	X	X	X	X
46	55	603.6968	603.6970	-0.33	SE**S**TEDQAME	2	X	X	X	X
46	55	643.6799	643.6802	-0.47	**S**E**S**TEDQAME	2			X	
46	56	661.2088	661.2105	-2.57	SE**S**TEDQAMED	2				X
46	58	821.7845	821.7832	1.58	**S**E**S**TEDQAMEDIK	2	X	X	X	X
47	49	336.1385	336.1401	-4.76	EST	1		X	X	X
48	54	431.1381	431.1384	-0.70	**S**TEDQAM	2	X	X	X	X
49	52	492.1915	492.1936	-4.27	TEDQ	1			X	X
49	54	347.6386	347.6393	-2.01	TEDQAM	2			X	X
50	52	391.1441	391.1460	-4.86	EDQ	1		X	X	
51	53	480.1746	480.1759	-2.71	DQA**M**	1			X	
55	57	376.1711	376.1714	-0.80	EDI	1	X	X	X	X
55	58	504.2649	504.2664	-2.97	EDIK	1			X	X
56	58	375.2233	375.2238	-1.33	DIK	1	X	X	X	X
62	74	841.2581	841.2578	0.36	AE**S**I**SSS**EEIVPN	2	X	X	X	
64	66	386.1315	386.1323	-2.07	SI**S**	1	X	X	X	X
64	74	661.2529	661.2517	1.81	SIS**SS**EEIVPN	2				X
64	74	701.2341	701.2348	-1.00	SI**SSS**EEIVPN	2	X	X	X	X
64	74	741.2181	741.2180	0.13	**S**I**SSS**EEIVPN	2	X	X	X	X
64	79	1026.868	1026.8663	1.66	SI**SSS**EEIVPN**S**VEQK	2	X	X	X	X
65	74	657.7179	657.7188	-1.37	I**SSS**EEIVPN	2	X	X	X	X
65	79	943.3653	943.3671	-1.91	I**SSS**EEIVPNSVEQK	2	X	X	X	X
65	79	983.3490	983.3503	-1.32	I**SSS**EEIVPN**S**VEQK	2	X	X	X	X
66	74	601.1766	601.1768	-0.33	**SSS**EEIVPN	2	X	X	X	
67	73	920.3035	920.3050	-1.63	**SS**EEIVP	1	X	X	X	
67	74	477.6941	477.6944	-0.63	**S**SEEIVPN	2	X	X	X	X
67	74	517.6769	517.6776	-1.35	**SS**EEIVPN	2	X	X	X	X
67	79	562.5417	562.5418	-0.18	**SS**EEIVPN**S**VEQK	3	X	X	X	X
68	74	787.3812	787.3832	-2.54	SEEIVPN	1	X	X	X	X
68	74	867.3469	867.3496	-3.11	**S**EEIVPN	1	X	X	X	X
69	74	700.3500	700.3512	-1.71	EEIVPN	1	X	X	X	X
69	79	676.3120	676.3107	1.92	EEIVPN**S**VEQK	2				X

70	72	360.2128	360.2129	-0.28	EIV	1	X	X	X	X
70	74	571.3086	571.3086	0.00	EIVPN	1	X	X	X	X
70	79	611.7877	611.7894	-2.78	EIVPN**S**VEQK	2	X	X	X	X
71	73	328.2232	328.2231	0.30	IVP	1	X	X	X	X
71	74	442.2652	442.2660	-1.81	IVPN	1	X	X	X	X
71	79	547.2685	547.2681	0.73	IVPN**S**VEQK	2	X	X	X	X
72	74	329.1810	329.1819	-2.73	VPN	1	X	X	X	X
74	77	528.1686	528.1701	-2.84	N**S**VE	1	X	X	X	X
75	79	590.3120	590.3144	-4.07	SVEQK	1			X	
75	79	670.2776	670.2807	-4.62	**S**VEQK	1	X	X	X	X
75	82	524.7437	524.7448	-2.10	**S**VEQKHIQ	2	X	X	X	X
80	82	397.2178	397.2194	-4.03	HIQ	1				X
80	88	526.7717	526.7722	-0.95	HIQKEDVPS	2				X
83	88	674.3335	674.3355	-2.97	KEDVPS	1	X	X	X	X
84	86	362.1572	362.1558	3.87	EDV	1		X	X	X
84	88	546.2391	546.2406	-2.75	EDVPS	1	X			
85	87	330.1648	330.1660	-3.63	DVP	1	X	X	X	X
85	88	417.1967	417.1980	-3.12	DVPS	1	X	X	X	X
85	89	546.2393	546.2406	-2.38	DVPSE	1				X
85	89	351.6738	351.6745	-1.99	DVPSER	2	X			
85	93	518.2590	518.2589	0.19	DVPSERYLG	2				X
89	97	585.7946	585.7931	2.56	ERYLGYLEQ	2			X	X
91	93	352.1857	352.1867	-2.84	YLG	1	X	X	X	X
91	94	515.2494	515.2500	-1.16	YLGY	1	X			
93	96	481.2283	481.2293	-2.08	GYLE	1	X	X	X	X
93	98	722.3719	722.3735	-2.21	GYLEQL	1			X	
93	100	496.2832	496.2822	2.01	GYLEQLLR	2				X
94	96	424.2074	424.2078	-0.94	YLE	1	X	X	X	X
94	98	665.3495	665.3505	-1.50	YLEQL	1	X	X	X	X
94	100	467.7727	467.7715	2.57	YLEQLLR	2			X	X
95	97	389.2020	389.2031	-2.83	LEQ	1	X	X	X	
97	99	373.2438	373.2445	-1.88	QLL	1	X	X	X	X
99	114	485.3043	485.3029	2.88	LRLKKYKVPQLEIVPN	4				X
100	102	416.2963	416.2980	-4.08	RLK	1			X	
103	108	381.7288	381.7291	-0.79	KYKVPQ	2	X	X	X	X
104	108	634.3555	634.3559	-0.63	YKVPQ	1	X	X	X	X
105	108	471.2926	471.2917	1.91	KVPQ	1			X	
105	110	357.2132	357.2127	1.40	KVPQLE	2			X	
105	114	568.8390	568.8373	2.99	KVPQLEIVPN	2			X	X
108	110	389.2026	389.2031	-1.28	QLE	1	X	X	X	

108	114	406.7297	406.7293	0.98	QLEIVPN	2			X	X
109	111	374.2288	374.2286	0.53	LEI	1	X	X	X	X
109	112	473.2970	473.2982	-2.54	LEIV	1			X	
109	114	684.3942	684.3927	2.19	LEIVPN	1	X	X	X	X
111	119	547.7476	547.7475	0.18	IVPN**S**AEER	2	X	X	X	X
115	120	671.2365	671.2396	-4.62	**S**AEER	1	X	X	X	X
115	122	421.2117	421.2118	-0.24	SAEERLH	2				X
115	122	461.1952	461.1949	0.65	**S**AEERLH	2	X	X	X	X
115	123	504.7095	504.7109	-2.77	**S**AEERLHS	2	X	X	X	X
115	123	570.2323	570.2312	1.93	**S**AEERLHSM	2				X
115	124	423.1881	423.1882	-0.24	**S**AEERLHSMK	3	X	X	X	X
117	119	433.2025	433.2041	-3.69	EER	1		X		
125	127	318.1650	318.1660	-3.14	EGI	1	X	X	X	X
141	143	332.1811	332.1816	-1.51	ELA	1		X	X	
147	149	358.1972	358.1973	-0.28	PEL	1	X	X	X	X
148	150	408.2138	408.2129	2.20	ELF	1	X	X	X	X
150	156	501.2649	501.2638	2.19	FRQFYQL	2			X	X
154	156	423.2230	423.2238	-1.89	YQL	1		X	X	X
166	169	491.2875	491.2864	2.24	YVPL	1	X	X	X	X
170	172	468.2068	468.2089	-4.49	GTQY	1		X	X	X
183	185	327.1647	327.1663	-4.89	PNP	1		X		
183	187	497.2709	497.2718	-1.81	PNPIG	1	X	X	X	X
197	199	415.2337	415.2340	-0.72	PLW	1				X
					αS2-casein-derived peptides					
6	18	876.2639	876.2662	-2.62	HV**SSS**EESII**S**QE	2	X	X	X	
6	21	1072.3683	1072.3692	-0.84	HV**SSS**EESII**S**QETYK	2	X	X	X	X
7	17	703.2309	703.2323	-1.99	V**SSS**EESIISQ	2	X	X	X	X
7	21	669.5622	669.5622	0.00	V**SSS**EESII**S**QETYK	3	X	X	X	X
8	13	785.1605	785.1638	-4.20	S**SS**EES	1	X	X	X	X
8	21	914.3218	914.3224	-0.66	S**SS**EESII**S**QETYK	2	X			
9	14	811.2148	811.2158	-1.23	**SS**EESI	1	X	X	X	X
9	16	546.1524	546.1528	-0.73	**SS**EE**S**IIS	2	X	X	X	
9	18	674.7017	674.7034	-2.52	**SS**EESII**S**QE	2	X	X	X	X
9	19	725.2276	725.2272	0.55	**SS**EESII**S**QET	2	X	X	X	X
9	21	870.8051	870.8063	-1.38	**SS**EESII**S**QETYK	2	X	X	X	
10	18	591.2036	591.2042	-1.01	**S**EESII**S**QE	2		X	X	
13	21	574.7645	574.7654	-1.57	SII**S**QETYK	2	X	X	X	X
14	16	332.2175	332.2180	-1.51	IIS	1	X	X	X	

14	16	412.1838	412.1843	-1.21	II**S**	1	X	X	X	X
14	18	335.1459	335.1464	-1.49	II**S**QE	2	X			
14	21	531.2489	531.2494	-0.94	II**S**QETYK	2	X			
17	21	334.6650	334.6661	-3.29	QETYK	2	X			
18	20	412.1701	412.1714	-3.15	ETY	1			X	
28	31	430.2296	430.2296	0.00	INPS	1	X	X	X	X
28	31	510.1943	510.1960	-3.33	INP**S**	1	X	X	X	X
28	33	384.1699	384.1704	-1.30	INP**S**KE	2	X	X	X	X
28	35	497.7330	497.7339	-1.81	INP**S**KENL	2	X	X	X	X
41	43	375.2223	375.2238	-4.00	EVK	1		X	X	X
42	44	346.1970	346.1973	-0.87	EVV	1	X	X	X	X
49	51	406.1441	406.1456	-3.69	EEE	1	X	X	X	
50	52	440.1662	440.1664	-0.45	EEY	1	X	X	X	X
54	61	518.1217	518.1215	0.39	IG**SSS**EES	2	X	X	X	X
56	63	1065.2049	1065.2098	-4.60	**SSS**EESAE	1	X	X	X	X
56	66	668.6849	668.6852	-0.45	**SSS**EESAEVAT	2	X			
56	70	911.3093	911.3094	-0.11	S**SS**EE**S**AEVATEEVK	2				X
58	65	981.2843	981.2850	-0.71	**S**EE**S**AEVA	1			X	X
67	69	376.1705	376.1714	-2.39	EEV	1	X	X	X	X
73	77	613.2915	613.2940	-4.08	VDDKH	1				X
100	114	604.9988	604.9967	3.47	YQGPIVLNPWDQVKR	3	X	X	X	X
100	115	643.0113	643.0110	0.47	YQGPIVLNPWDQVKRN	3				X
101	103	301.1492	301.1506	-4.65	QGP	1		X	X	
101	114	550.6429	550.6423	1.09	QGPIVLNPWDQVKR	3				X
102	106	498.3301	498.3286	3.01	GPIVL	1	X	X		
104	106	344.2549	344.2544	1.45	IVL	1	X	X	X	X
104	109	371.2192	371.2183	2.42	IVLNPW	2	X			
104	110	856.4568	856.4563	0.58	IVNLPWD	1				X
104	114	456.5991	456.5980	2.41	IVLNPWDQVKR	3				X
109	111	448.1821	448.1827	-1.34	WDQ	1	X	X	X	X
103	105	328.2232	328.2231	0.30	PIV	1	X	X	X	X
115	122	812.4497	812.4512	-1.85	NAVPITPT	1				X
116	122	349.7074	349.7078	-1.14	AVPITPT	2	X	X	X	X
116	123	406.2508	406.2498	2.46	AVPITPTL	2				X
118	122	528.3034	528.3028	1.14	PITPT	1	X	X	X	X
119	122	431.2487	431.2500	-3.01	ITPT	1	X	X	X	X
119	123	544.3349	544.3341	1.47	ITPTL	1			X	X
123	128	386.7185	386.7192	-1.81	LNREQL	2	X	X	X	X
128	130	320.1809	320.1816	-2.19	LST	1	X	X	X	X
132	134	391.1444	391.1460	-4.09	EEN	1	X	X	X	

139	142	493.1954	493.1963	-1.82	VDME	1	X	X	X	X
139	147	568.7155	568.7145	1.76	VDME**S**TEVF	2				X
140	142	394.1264	394.1279	-3.81	DME	1	X	X	X	X
144	146	348.1763	348.1765	-0.57	TEV	1		X	X	X
145	148	495.2455	495.2449	1.21	EVFT	1	X	X	X	X
146	148	366.2018	366.2023	-1.37	VFT	1	X	X	X	X
150	155	360.2177	360.2185	-2.22	KTKLTE	2				X
155	157	534.2395	534.2406	-2.06	EEEK	1		X		
156	158	405.1965	405.1980	-3.70	EEK	1	X	X	X	X
159	163	332.1817	332.1823	-1.81	NRLNF	2	X			
160	162	402.2440	402.2459	-4.72	RLN	1	X	X	X	X
161	163	393.2139	393.2132	1.78	LNF	1	X	X	X	X
163	165	407.2638	407.2653	-3.68	FLK	1	X	X	X	X
173	175	352.2099	352.2105	-1.70	KFALPQ	2		X		
174	178	575.3198	575.3188	1.74	FALPQ	1			X	X
179	181	423.2595	423.2602	-1.65	YLK	1	X	X	X	X
182	184	382.1959	382.1973	-3.66	TVY	1	X	X	X	X
193	196	543.2945	543.2926	3.50	WIQP	1			X	
193	197	336.1974	336.1974	0.00	WIQPK	2	X	X	X	X
194	197	485.3085	485.3082	0.62	IQPK	1			X	
198	203	360.7184	360.7182	0.55	TKVIPY	2	X	X	X	X
198	207	417.9209	417.9202	1.67	TKVIPYVRYL	3		X	X	X
199	203	619.3810	619.3814	-0.65	KVIPY	1	X	X	X	X
200	203	491.2869	491.2864	1.02	VIPY	1	X	X	X	X
200	207	511.8067	511.8053	2.74	VIPYVRYL	2	X			X
202	204	534.3034	534.3035	-0.19	PYVR	1		X		
203	207	357.2029	357.2027	0.56	YVRYL	2	X	X	X	X

One code letter was used for amino acids.
Underlined letter means phosphorylated amino acid.
z means peptide charge
