# Supplementary material for: An Integrated Peptidomics and In Silico Approach to Identify Novel Anti-Diabetic Peptides in Parmigiano-Reggiano Cheese
Source: Biology (Basel). 2021 Jun 21;10(6):563. doi: 10.3390/biology10060563 (PMC8234620; doi:10.3390/biology10060563)
Supplement: Supplementary file 1 [file biology-10-00563-s001.zip › Table S2.pdf]

Table S2. Potential bioactive peptides (threshold > 0.5) identified though PeptideRanker software. Peptides are sorted in decreasing peptide rank values.

Peptide rank value	Sequence
0.988634	PPF
0.983520	PFP
0.979835	PLW
0.977357	PFL
0.967512	FFV
0.929380	PFPI
0.919188	VPPFL
0.907515	FVAPFP
0.906948	VMF
0.888252	IHPF
0.875432	FFVAP
0.852911	FGK
0.851491	PGPIP
0.845170	IPPL
0.836815	FVAPFPEVFGK
0.835842	LNF
0.821039	VAPFP
0.820430	FVAPFPEVF
0.818475	GPVRGPFPIIV
0.809221	NIPPL
0.805333	FLK
0.798251	VVPPFL
0.797737	LVYPFP
0.796828	PGPI
0.794971	LPLP
0.793557	WIQP
0.774469	YQEPVLGPVRGPFPIIV
0.770664	KIHPF
0.768503	VAPFPEVFGK
0.767686	EMPFPK
0.766829	IPP
0.766282	PGPIPN
0.763061	FRQFYQL
0.760743	VFG
0.756965	EPF
0.756225	VAPFPEVF
0.753363	SLVYPFPGPIPN

0.751927	PIP
0.750635	LVYPFPGP
0.747983	VVPPF
0.742455	PFPE
0.715773	APFPE
0.694061	PNP
0.691626	GPIPN
0.686943	DKIHPF
0.672852	QGP
0.672533	LHLPLP
0.661305	NRLNF
0.659136	FALPQ
0.657245	QGPIVLNPWDQVKR
0.657222	PNPIG
0.655570	FVAPFPE
0.650217	WIQPK
0.646746	LVYPFPGPIPN
0.642316	RFFVAPFPE
0.642092	WDQ
0.640399	YLG
0.632981	FVAPFPEVFGKE
0.622409	VVVPPFL
0.613770	YLGY
0.613611	PIPN
0.609408	IPPLT
0.608844	FVAPFPEVFGKEK
0.608013	FVAP
0.607572	NENLLRF
0.605988	TPVVVPPFL
0.592346	YQGPIVLNPWDQVKRN
0.592092	FVAPFPEVFGKEKVNELSK
0.590640	RPKHPIKHQGLPQEVLNENLLRF
0.588075	LPPT
0.584709	QTPVVVPPFL
0.573178	ENLLRF
0.568648	ELF
0.561996	FVAPFPEVFGKEKVN
0.561001	YQGPIVLNPWDQVKR
0.542884	GLPQ
0.542672	VVVPPF

0.536380	IVLNPW
0.518940	VIPYVRYL
0.515736	KFQ
0.514265	VPP
0.502974	GPIVL
0.501594	LLY

Threshold was set at 0.5. Peptides with a peptide rank value below 0.5 are not shown
