# Supplementary material for: An Integrated Peptidomics and In Silico Approach to Identify Novel Anti-Diabetic Peptides in Parmigiano-Reggiano Cheese
Source: Biology (Basel). 2021 Jun 21;10(6):563. doi: 10.3390/biology10060563 (PMC8234620; doi:10.3390/biology10060563)
Supplement: Supplementary file 1 [file biology-10-00563-s001.zip › Table S3.pdf]

Table S3. Selection of potential DPPIV-inhibitory peptides as a function of PepSite *P* value, structure-activity relationship (SAR) modelling and *in silico* gastro-intestinal digestion (GID) stability. Selected peptides were reported in bold red.

Sequence	PepSite *P* value for DPPIV (pdb: 1NU6)	SAR	GID
PPF	1.63E-05	/	/
PNP	2.64E-05	/	/
**IPP**	4.26E-05	I at the N-terminus and P in second position	Stable
QGP	1.02E-04	/	/
PFP	1.11E-04	/	/
PIP	1.18E-04	/	/
VPP	1.59E-04	/	/
**LPPT**	4.76E-04	L at the N-terminus and P in second position	Stable
PFPE	5.59E-04	/	/
QTPVVVPPFL	6.76E-04	/	/
PGPI	7.04E-04	/	/
KIHPF	7.71E-04	/	/
RFFVAPFPE	8.09E-04	/	/
FALPQ	8.71E-04	F at the N-terminus and A in second position	Unstable. Potential cleavage sites: F-A chymotrypsin and pepsin; L-P pepsin
FVAPFPE	9.62E-04	/	/
FVAPFP	1.02E-03	/	/
PFL	1.13E-03	/	/
GPIPN	1.20E-03	/	/
**APFPE**	1.26E-03	A at the N-terminus and P in second position	Stable
VAPFPEVFGK	1.31E-03	/	/
VMF	1.33E-03	/	/
PNPIG	1.47E-03	/	/
PGPIP	1.59E-03	/	/

IPPL*	1.68E-03	I at the N-terminus and P in second position	Stable
VPPFL	1.84E-03	/	/
VVPPF	1.84E-03	/	/
LVYPFPGP	1.86E-03	/	/
FGK	2.02E-03	/	/
PGPIPN	2.07E-03	/	/
IPPLT	2.20E-03	I at the N-terminus and P in second position	Unstable. Potential cleavage sites: L-T chymotrypsin
FRQFYQL	2.46E-03	/	/
WIQPK*	2.48E-03	/	/
VAPFP	2.64E-03	/	/
FVAPFPEVF	2.83E-03	/	/
LHLPLP	2.89E-03	/	/
DKIHPF	2.91E-03	/	/
FFVAP	2.96E-03	/	/
EPF	3.45E-03	/	/
PFPI	3.47E-03	/	/
VAPFPEVF	3.61E-03	/	/
PIPN	3.62E-03	/	/
IHPF	3.80E-03	/	/
NIPPL	3.84E-03	/	/
PLW	4.31E-03	/	/
EMPFPK	4.91E-03	/	/
FFV	4.98E-03	/	/
TPVVVPPFL	5.22E-03	/	/
WDQ	5.28E-03	/	/
KFQ	5.37E-03	/	/
VFG	5.47E-03	/	/
FVAP	5.54E-03	/	/

LPLP	6.04E-03	L at the N-terminus and P in second position	Unstable. Potential cleavage sites: L-P pepsin
LVYPFP	6.28E-03	/	/

* Previously identified as DPPIV-inhibitor

Threshold was set at 0.01. Peptides with *P*-value below the threshold are not shown
