# Supplementary material for: An Integrated Peptidomics and In Silico Approach to Identify Novel Anti-Diabetic Peptides in Parmigiano-Reggiano Cheese
Source: Biology (Basel). 2021 Jun 21;10(6):563. doi: 10.3390/biology10060563 (PMC8234620; doi:10.3390/biology10060563)
Supplement: Supplementary file 1 [file biology-10-00563-s001.zip › Table S4.pdf]

Table S4. Selection of potential α-glucosidase-inhibitory peptides on the basis on the PepSite *P* value, structure-activity relationship (SAR) modelling and *in silico* gastro-intestinal digestion (GID) stability. Peptides were ranked on the basis of the PepSite *P* value. Selected peptides were reported in bold red.

Sequence	*PepSite P value for α-glucosidase (pdb: 5NN3)*	*SAR*	*GID*
PNP	1.33E-04	/	/
IPP	1.55E-04	/	/
PFP*	1.75E-04	/	/
**PPF**	1.93E-04	F at the C-terminus and P at the penultimate position	Stable
QGP	2.47E-04	/	/
PFPI	2.58E-04	/	/
VPP	3.48E-04	/	/
PGPIP	3.65E-04	/	/
IPPL	4.15E-04	/	/
LPPT	4.23E-04	/	/
VAPFP	4.48E-04	/	/
APFPE	4.48E-04	/	/
PGPI	4.56E-04	/	/
PNPIG	4.65E-04	/	/
PIPN	4.71E-04	/	/
EMPFPK	5.06E-04	/	/
PFPE	5.80E-04	/	/
FVAPFPE	6.39E-04	/	/
FVAPFP	7.41E-04	/	/
LPLP	7.56E-04	/	/
WIQP	8.20E-04	/	/
KIHPF	8.26E-04	K at the N-terminus, F at the C-terminus and P at the penultimate position	Unstable. Potential cleavage sites: K-I trypsin; P-F pepsin
IHPF	8.50E-04	F at the C-terminus and P at the penultimate position	Unstable. Potential cleavage sites: P-F pepsin
WIQPK	8.51E-04	/	/
NIPPL	9.27E-04	/	/

IPPLT	9.27E-04	/	/
GLPQ	1.00E-03	/	/
GPIPN	1.05E-03	/	/
VPPFL	1.07E-03	/	/
**VVPPF**	1.07E-03	F at the C-terminus and P at the penultimate position	Stable
DKIHPF	1.19E-03	F at the C-terminus and P at the penultimate position	Unstable. Potential cleavage sites: K-I trypsin; P-F pepsin
VAPFPEVF	1.32E-03	/	/
PGPIPN	1.39E-03	/	/
FRQFYQL	1.40E-03	/	/
**VVVPPF**	1.50E-03	F at the C-terminus and P at the penultimate position	Stable
LVYPFP	1.50E-03	/	/
VVPPFL	1.50E-03	/	/
FALPQ	1.51E-03	/	/
RFFVAPFPE	1.92E-03	/	/
FVAPFPEVF	1.92E-03	/	/
KFQ	2.48E-03	/	/
VAPFPEVFGK	2.62E-03	/	/
PFL	2.82E-03	/	/
LVYPFPGP	3.53E-03	/	/
FVAP	3.56E-03	/	/
PIP	3.60E-03	/	/
VIPYVRYL	4.29E-03	/	/
LHLPLP	4.34E-03	/	/
VVVPPFL	5.51E-03	/	/
PLW	5.69E-03	/	/
EPF	5.94E-03	F at the C-terminus and P at the penultimate position	Unstable. Potential cleavage sites: E-P pepsin
FGK	6.65E-03	/	/
NRLNF	6.90E-03	/	/
FFVAP	8.18E-03	/	/

IVLNPW	8.39E-03	/	/
QTPVVVPPFL	9.33E-03	/	/

* Previously identified as α-glucosidase-inhibitor

Threshold was set at 0.01. Peptides with *P*-value below the threshold are not shown
