# Supplementary material for: An Integrated Peptidomics and In Silico Approach to Identify Novel Anti-Diabetic Peptides in Parmigiano-Reggiano Cheese
Source: Biology (Basel). 2021 Jun 21;10(6):563. doi: 10.3390/biology10060563 (PMC8234620; doi:10.3390/biology10060563)
Supplement: Supplementary file 1 [file biology-10-00563-s001.zip › Table S5.pdf]

Table S5. PepSite *P* value for α-amylase. Peptides were ranked on the basis of the PepSite *P* value.

Sequence	PepSite P value for α-amylase (pdb: 1PIG)	SAR	GID
PNP	1.37E-04	/	/
PPF	1.79E-04	/	/
IPP	2.37E-04	/	/
NIPPL	3.36E-04	/	/
QGP	3.54E-04	/	/
FALPQ	4.05E-04	/	/
VPP	4.14E-04	/	/
PNPIG	5.92E-04	/	/
IPPL	6.74E-04	/	/
LPPT	7.94E-04	/	/
PFL	9.59E-04	/	/
VPPFL	1.01E-03	/	/
LHLPLP	1.02E-03	/	/
WIQP	1.03E-03	/	/
LPLP	1.04E-03	/	/
VVVPPF	1.07E-03	/	/
VVPPF	1.08E-03	/	/
GLPQ	1.19E-03	/	/
FVAP	1.46E-03	/	/
IPPLT	1.52E-03	/	/
VAPFP	1.57E-03	/	/
LVYPFP	1.77E-03	/	/
EMPFPK	2.03E-03	/	/
FFVAP	2.07E-03	/	/
WIQPK	2.34E-03	/	/
RFFVAPFPE	2.34E-03	/	/
PLW	2.52E-03	/	/
PGPIPN	2.53E-03	/	/
APFPE	2.70E-03	/	/
VVPPFL	3.04E-03	/	/
WDQ	3.05E-03	/	/
DKIHPF	3.24E-03	/	/
GPIPN	3.55E-03	/	/
KFQ	3.75E-03	/	/
VVVPPFL	4.11E-03	/	/
PGPI	4.31E-03	/	/

KIHPF	4.31E-03	/	/
PIPN	4.66E-03	/	/
IVLNPW	5.34E-03	/	/
EPF	5.51E-03	/	/
PFPI	5.62E-03	/	/
VIPYVRYL	6.20E-03	/	/
NRLNF	6.63E-03	/	/
GPIVL	7.49E-03	/	/
PFP	7.81E-03	/	/
PIP	7.81E-03	/	/
NENLLRF	8.48E-03	/	/
FRQFYQL	8.52E-03	/	/
FVAPFPE	9.19E-03	/	/
FGK	9.66E-03	/	/
PGPIP	9.92E-03	/	/

Threshold was set at 0.01. Peptides with *P*-value below the threshold are not shown
